# Incremental benefit in correlation with histology of native T1 mapping, partition coefficient and extracellular volume fraction in patients with aortic stenosis

**DOI:** 10.1186/1532-429X-18-S1-O48

**Published:** 2016-01-27

**Authors:** Vassilis Vassiliou, Katharina Wassilew, Tamir Malley, Claire E Raphael, Rebecca S Schofield, Kevin Kirby, Alex D Bowman, Karen Symmonds, Bruce S Spottiswoode, Andreas Greiser, Iain Pierce, David Firmin, Peter Gatehouse, Dudley J Pennell, Sanjay Prasad

**Affiliations:** 1 CMR, Royal Brompton Hospital, London, UK; 2 National Heart and Lung Institute, Imperial College London, London, UK; 3 Cardiac Pathology Unit, Department of Cardiothoracic and Vascular Surgery, Deutsches Herzzentrum Berlin, Berlin, Germany; 4 Imaging, UCL, London, UK; 5 Siemens Healthcare, Erlangen, Germany; 6 Siemens Medical Solutions USA, Inc, Chicago, IL USA; 7 Department of Histopathology, Royal Brompton Hospital, London, UK

## Background

We investigated the histological correlation of native T1 maps, partition coefficient and extracellular volume fraction (ECV) using an 11 heart beat (11 HB) MOLLI for identification of overall burden of fibrosis.

## Methods

Ten patients (8 male, age 73 ± 7 years; all in sinus rhythm, 2 with ventricular ectopy) with severe aortic stenosis (3 with coexisting coronary artery disease) scheduled for surgical aortic valve replacement underwent CMR on a 1.5T scanner (MAGNETOM Avanto, Siemens Healthcare, Erlangen). The 11HB MOLLI sequence (Siemens investigational prototype WIP 448B) was acquired before and 15 minutes post 0.1 mmol/kg gadolinium administration. Incorporating hematocrit results from the same day. This allowed native T1 maps, partition coefficient and ECV calculation. Images were obtained twice at end diastole at basal, and twice at mid left ventricular level. The average of all measurements was used to calculate ECV using the standard formula Partition Coefficient= [(1/T1myocardium post contrast-1/T1 myocardium native)]/[(1/T1 blood post contrast-1/T1 blood native)] with x(1-HCt) for ECV. Similar regions of interest were drawn in the septum at both levels for T1 values. Intraoperatively, trucut biopsies were taken from the left ventricular apical anterior/ lateral wall through the epicardium to allow histological characterization of the full myocardial wall, and fixed in warm buffered formalin. Histological analysis of formalin-fixed paraffin-embedded, transmural myocardial biopsies of the left ventricle was performed on hematoxylin/eosin and Picrosirius red-stained 3-micron-thick sections by a blinded experienced cardiac pathologist. Images were analysed using a purpose-built software (Nikon NIS elements BR) on a NIKON Eclipse light projection microscope to determine the extent of overall and reactive interstitial fibrosis, which was expressed as collagen volume fraction (%) per square millimetre.

## Results

Native T1 mapping, partition coefficient and ECV all correlated with histologically measured fibrosis. However, native T1 mapping showed the least accuracy (panel A, R^2^ = 0.42) and ECV showed the highest accuracy (panel B, R^2^ = 0.83). Partition coefficient was more accurate than native T1 mapping but only very marginally less so than ECV (panel C, R^2^ = 0.80).

## Conclusions

These results suggest that native T1 mapping is less accurate than partition coefficient and ECV for overall fibrosis. Therefore, post gadolinium images to enable calculation of partition coefficient and ECV should be routinely obtained to increase accuracy.

**Figure 1:**
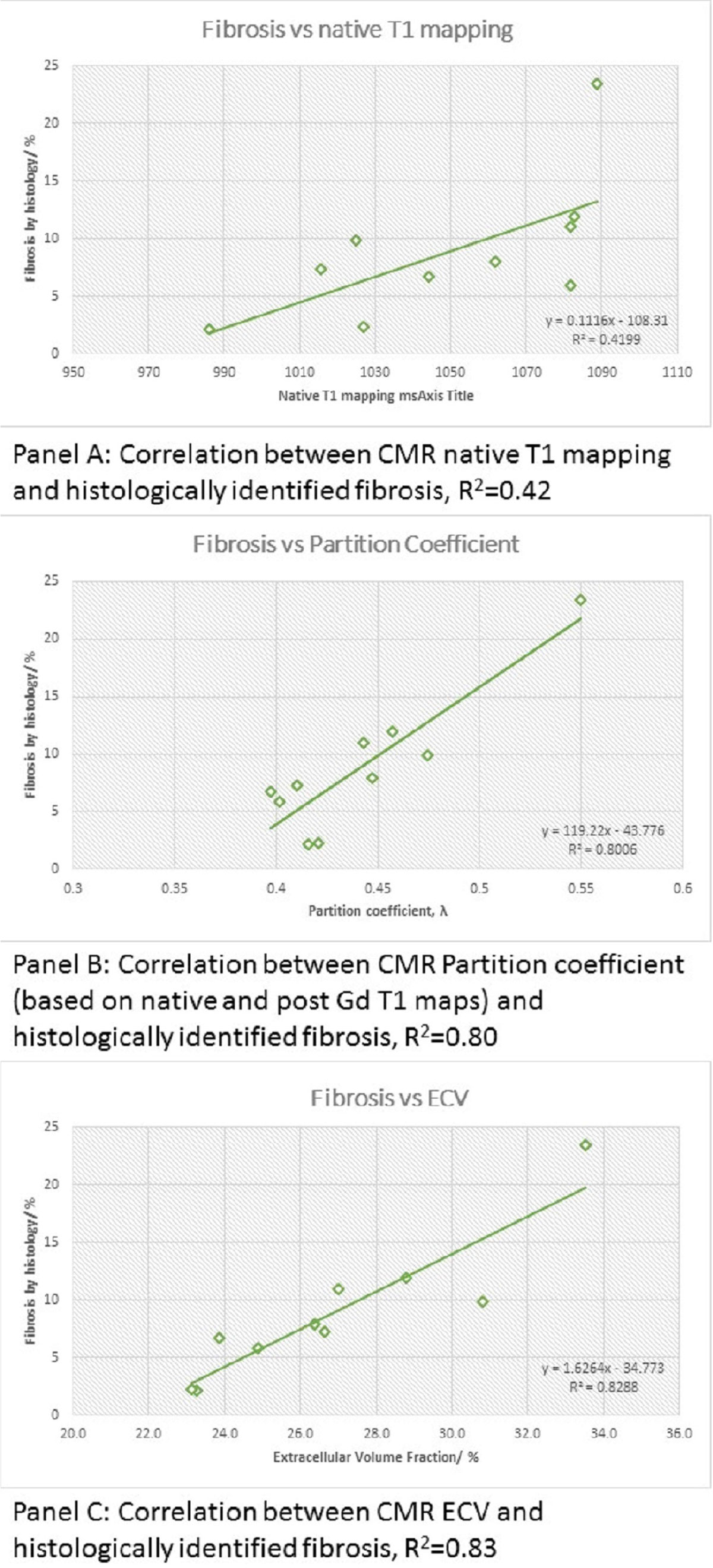
**Correlation between native T1 and histological fibrosis (Panel A, top); correlation between partition coefficient and and histological fibrosis (Panel B, middle); and correlation between ECV and histological fibrosis (panel C, bottom)**. Both partition coefficient and ECV perfomred better than native T1 maps alone.

